# Shell field morphogenesis in the polyplacophoran mollusk *Acanthochitona rubrolineata*

**DOI:** 10.1186/s13227-023-00209-9

**Published:** 2023-04-06

**Authors:** Yuxiu Xia, Pin Huan, Baozhong Liu

**Affiliations:** 1 CAS and Shandong Province Key Laboratory of Experimental Marine Biology, Institute of Oceanology, Chinese Academy of Sciences, 7 Nanhai Road, Qingdao, 266071 China; 2 Laboratory for Marine Biology and Biotechnology, Pilot National Laboratory for Marine Science and Technology (Qingdao), Qingdao, China; 3 University of Chinese Academy of Sciences, Beijing, 100039 China

**Keywords:** Polyplacophoran, Mollusk, Shell field, Plate field, Morphogenesis, *Engrailed*

## Abstract

**Background:**

The polyplacophoran mollusks (chitons) possess serially arranged shell plates. This feature is unique among mollusks and believed to be essential to explore the evolution of mollusks as well as their shells. Previous studies revealed several cell populations in the dorsal epithelium (shell field) of polyplacophoran larvae and their roles in the formation of shell plates. Nevertheless, they provide limited molecular information, and shell field morphogenesis remains largely uninvestigated.

**Results:**

In the present study, we investigated shell field development in the chiton *Acanthochitona rubrolineata* based on morphological characteristics and molecular patterns. A total of four types of tissue could be recognized from the shell field of *A. rubrolineata*. The shell field comprised not only the centrally located, alternatively arranged plate fields and ridges, but also the tissues surrounding them, which were the precursors of the girdle and we termed as the girdle field. The girdle field exhibited a concentric organization composed of two circularly arranged tissues, and spicules were only developed in the outer circle. Dynamic *engrailed* expression and F-actin (filamentous actin) distributions revealed relatively complicated morphogenesis of the shell field. The repeated units (plate fields and ridges) were gradually established in the shell field, seemingly different from the manners used in the segmentation of *Drosophila* or vertebrates. The seven repeated ridges also experienced different modes of ontogenesis from each other. In the girdle field, the presumptive spicule-formation cells exhibited different patterns of F-actin aggregations as they differentiate.

**Conclusions:**

These results reveal the details concerning the structure of polyplacophoran shell field as well as its morphogenesis. They would contribute to exploring the mechanisms of polyplacophoran shell development and molluscan shell evolution.

**Supplementary Information:**

The online version contains supplementary material available at 10.1186/s13227-023-00209-9.

## Background

**Fig. 1:**
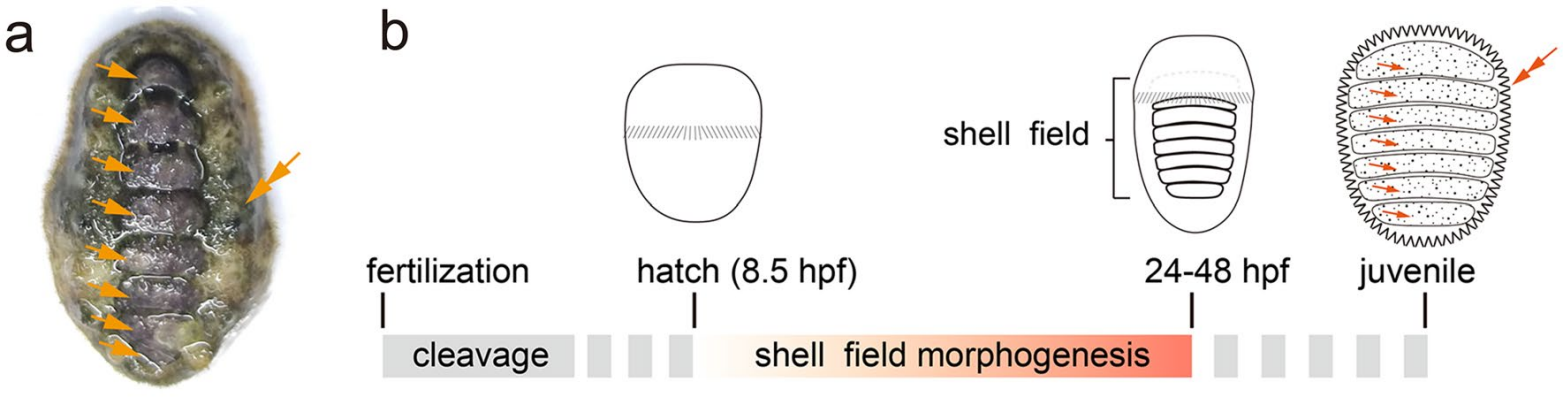
Polyplacophoran shell plates and their development. **a** Adult *A. rubrolineata*; dorsal view with the anterior to the top. The eight serially arranged shell plates (arrows) are encircled by the girdle (double arrow). **b** Generalized scheme depicting polyplacophoran shell ontogenesis. In some species, the newly hatched trochophore larvae do not have a recognizable shell field. After metamorphosis, seven calcified shell plates emerge in the juvenile (arrows) and spicules develop in the girdle (the double arrow). Developmental stages are referred to *A. rubrolineata*

Polyplacophora, whose extant members are called chitons, represents a unique lineage of mollusks. In particular, polyplacophorans possess eight shell plates aligned in a serial pattern along the anteroposterior axis [1] (Fig. 1a). This characteristic is emphasized due to its uniqueness in shell plate number compared to conchiferan mollusks (mostly having one or two shells; e.g., bivalves and gastropods) and its apparent (albeit limited) similarities with segmentation in animals, such as arthropods, annelids and vertebrates [2–5]. In addition, polyplacophorans also develop spicules (or sclerites/scales) in the tissue surrounding shell plates (girdle). As another type of mineralized structures, spicules are proposed to be evolutionarily and structurally related to shell plates and are informative to infer molluscan evolution [6–8].

In development, seven serially arranged units of tissues are formed on the dorsal epithelium of polyplacophoran larvae, which then produce seven shell plates after metamorphosis [9–11]; the eighth shell plate forms later. Each of the seven units that will secrete a shell plate is termed as a plate field [2, 6, 11]. We follow this terminology and further suggest that as in conchiferans, the total larval tissue of polyplacophorans that are related to shell formation should be termed as the shell field. This is not an evolutionary definition but is a developmental/functional one. We think the common terminology of the larval shell-formation tissues in conchiferans and polyplacophorans would ensure better comparisons among these shelled mollusks. As shown below, we indeed found taking all shell-formation tissue as a whole was important in certain contexts, e.g., those concerning shell formation mechanisms or evolutionary issues.

The polyplacophoran shell fields contain distinct cell populations, with different roles in the formation of shell plates and the connective tissues [6, 11]. On the molecular level, it has been revealed a number of genes showing striped expression in the shell field, including the well-accepted molluscan shell-formation gene *engrailed* [12], key developmental regulators *hox* genes [13–15] and others [16, 17]. Nevertheless, it is largely unknown what specific cell types these genes are expressed in. It seems the only exception is *engrailed*, which is expressed in cells not involved in shell plate secretion [12]. While the fates of cell populations inside the shell field are generally determined [6, 11], additional molecular information is required to elucidate the roles of related cells.

Given the distinct cell populations in different compositions of the shell field [6, 11], it is necessary to further ask how these cells are specified and how they are organized into the characteristic pattern. Nevertheless, current knowledge regarding shell field morphogenesis is limited except for a few morphological observations [9–11]. As mentioned above, calcified shell plates are only developed after metamorphosis and there is merely a shell field in larval stages [4, 11] (Fig. 1b). Meanwhile, a recognizable shell field is yet not formed in the newly hatched larvae of some species [10]. These facts indicate that shell field morphogenesis occurs during the period between hatching and metamorphosis (Fig. 1b). Indeed, the appearances of the shell field continuously change with larval development in *Rhyssoplax olivacea* (= *Chiton olivaceus*) [4], and gene expression in the shell field exhibits dynamic patterns during polyplacophoran larval development [13, 14, 16].

The morphogenesis of shell field establishes the framework for subsequent shell plate secretion, and thus investigations of this process would be essential to understand polyplacophoran shell formation. Moreover, given that development often provides important evolutionary implications, investigating the early development of polyplacophoran shell field may also provide clues to explore evolutionary issues, such as the origin of shell plates, the evolutionary relationships among the sclerotized structures of mollusks/spiralians, and even the evolution of animal segmentation [5, 7, 8, 18]. To explore more information about polyplacophoran shell development, in the present study, we investigated shell field morphogenesis in the chiton *Acanthochitona rubrolineata*. The results revealed details regarding the molecular patterns of different tissues inside the shell field, as well as those concerning their morphogenesis. These results add to the knowledge of polyplacophoran shell development and provide insights into molluscan shell evolution.

## Results

### General development of *A. rubrolineata*

**Fig. 2:**
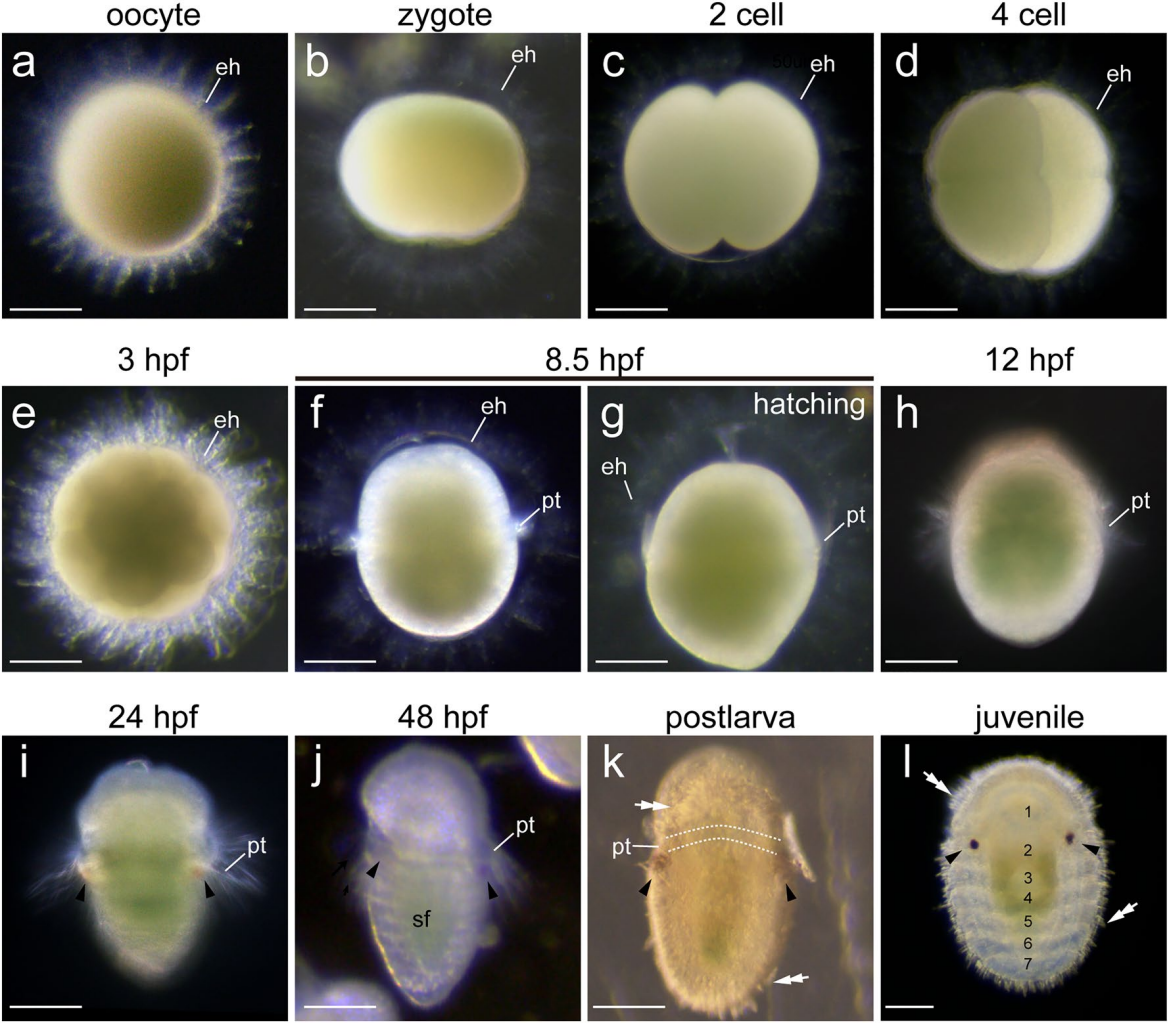
General development of *A. rubrolineata*. An egg hull (eh) can be recognized before the hatching of the trochophore larva **a–g**. In **b**, the zygote is dividing. Panel **h** shows an early trochophore. Larval eyes emerge at around 24 hpf (arrowheads in **i**), and are retained after metamorphosis (arrowheads in** k–l**). In **j**, the shell field (sf) showing serially arranged repeated units could be clearly recognized. Spicules (double arrows in **k** and **l**) start to develop during metamorphosis and seven shell plates could be observed in juveniles (numbers in **l**). pt, prototroch. Bars represent 50 μm

*A. rubrolineata* is a free spawning species, which directly releases sperm and oocytes into the water column. As in many chitons, a key feature of *A. rubrolineata* oocyte is that it is enclosed by an elaborate egg hull showing extensive superficial protrusions (Fig. 2a). After fertilization, zygotes experienced two rounds of equal cleavage followed by characterized spiral cleavage (Fig. 2b–e). Embryonic development occurred in the egg hull and only larval cilia (prototroch) could be discriminated during this period (Fig. 2f). Beginning at 8.5 h post fertilization (hpf), early trochophore larvae hatched by breaking through the egg hull (Fig. 2g, h). The times of hatching varied for different individuals (could be later than 10 hpf), but we did not observe evident changes in developmental rates between these individuals. Some individuals could not hatch due to abnormal development, and they could remain alive in the egg hull for several days.

At around 24 hpf, two larval eyes emerged in ventral lateral tissues of the posttrochal region (Fig. 2i), contrasting to the pretrochal eyes of conchiferan mollusks. Characteristic larval structures including a ventral foot anlagen and a dorsal shell field also developed (Fig. 2j). The subsequent larval development showed minor morphological changes under ordinary microscopy, except that the larval body gradually got elongated and somewhat flattened. Beginning at as early as 60 hpf, the larvae could metamorphose when proper substrates were supplied (Fig. 2k). However, if no inductive clues were available, they could remain as swimming larvae till at least 7 day post-fertilization without losing the capacity to metamorphose. During metamorphosis, the larvae greatly flattened their bodies and lost the prototroch. Spicules and seven shell plates were developed in marginal and central regions of the dorsal epithelium, respectively; the larval eyes were retained (Fig. 2l). Juveniles were morphologically similar with an adult chiton, despite the lack of the eighth shell plate (Fig. 2l).

Based on the aforementioned results, we concluded that very early larvae did not possess a recognizable shell field in *A. rubrolineata*, and the shell field was fully developed at 48 hpf (Fig. 2j). We thus used samples between 12 and 48 hpf in subsequent analyses.

### Shell field morphogenesis: morphological changes

**Fig. 3:**
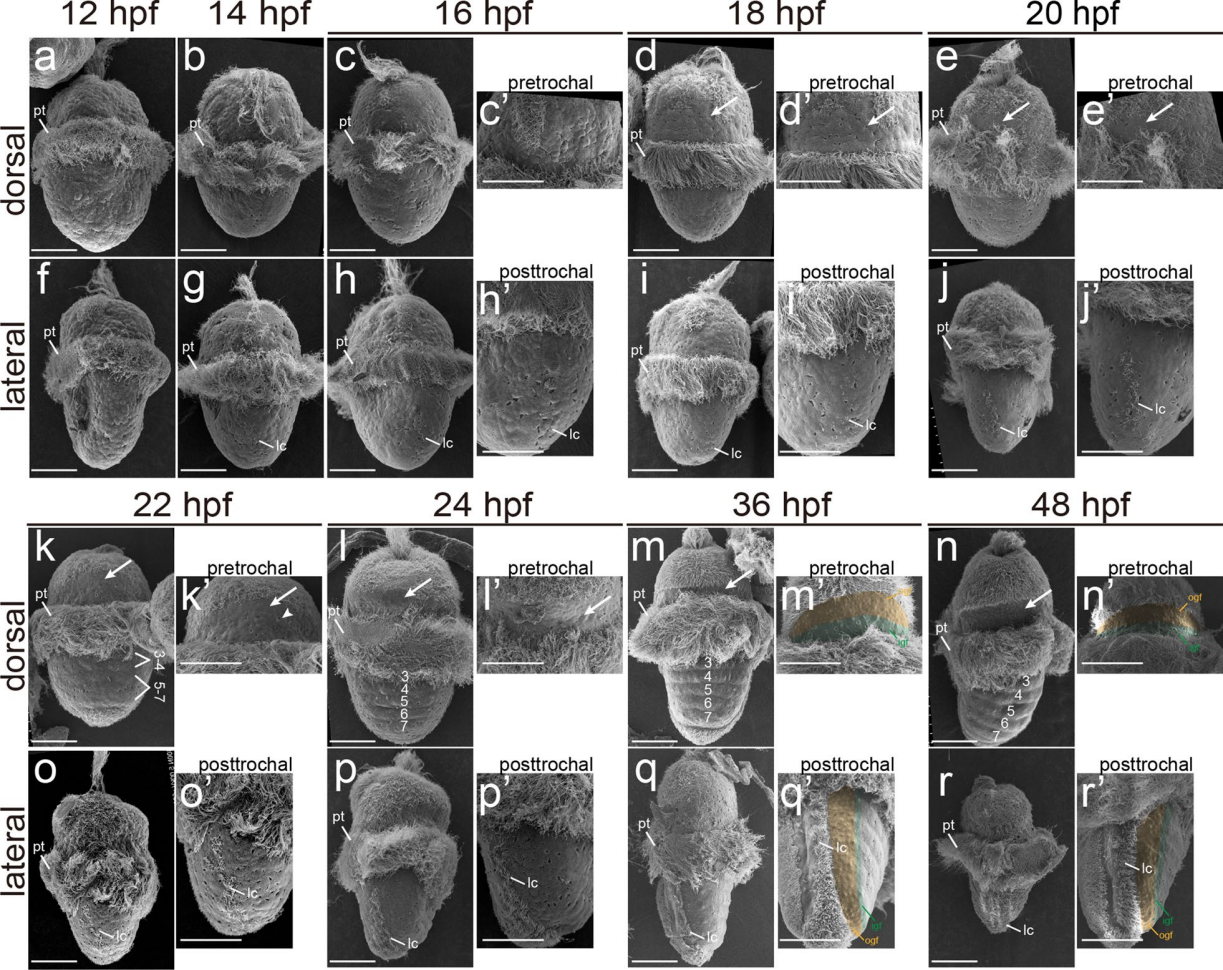
Larval development under SEM. Dorsal and lateral views are shown, with the anterior to the top. In early larvae (**a–c**, **f–h**), no differentiation of dorsal cells could be observed. For larvae after 16 hpf, panels **c’–e’, h’–j’, k’–r’** show the details of pretrochal and posttrochal regions. Note that for some stages they are not the same larvae as those in **c–e, h–j, k–r.** The pretrochal region of the shell field is indicated by arrows in **d–e** and **k–n**. The small pore in the cells adjacent to the prototroch are highlighted by the arrowhead in **k’**. In larvae after 24 hpf, alternative bulges and grooves are discernable (indicated by numbers in **l–n**). Outer girdle field (ogf) and inner girdle field (igf) exhibit different morphological characters after 36 hpf, and are indicated by brown and green shadows, respectively (**m’**, **n’**, **q’** and **r’**). lc, lateral cilia; pt, prototroch. Bars represent 50 μm

We first explored morphological changes using scanning electron microscopy (SEM). The early larvae at 12–16 hpf showed no recognizable dorsal structures (Fig. 3a–c, f–h). At 18 hpf, the cilia in the pretrochal region expanded compared to earlier larvae, leaving a non-ciliated area recognizable (Fig. 3d, d’). This region was subsequently revealed to be a part of the shell field. Beginning at 22 hpf, tissues inside in this region started to exhibit varied morphological characteristics (Fig. 3k, l). The inner tissues adjacent to the prototroch showed small pores on their surface (also observed in most posttrochal tissues; Fig. 3k’). At 36 and 48 hpf, these inner tissues developed irregularly distributed, shallow depressions on their surface (Fig. 3m’, n’). At the same time, the outer tissues in the pretrochal region, although showing no morphological characteristics in earlier stages, developed tiny protrusions (Fig. 3m’, n’). This morphological difference indicates the formation of two cell populations inside the pretrochal region.

The lateral cilia in the posttrochal region could be discerned at 14 hpf (Fig. 3g). Very close to these cilia were shell field tissues, which, however, showed no morphological characteristics in early larvae (Fig. 3h’–j’, o’, p’). At 36 and 48 hpf, this part of tissues developed tiny superficial protrusions similar to those of the outer tissues in the pretrochal region (Fig. 3q’, r’, compare to Fig. 3m’, n’). These larval tissues with tiny protrusions overall formed a circle enclosing the central region (Fig. 3m’, n’, q’, r’). Inside to these tissues, we revealed another type of circularly arranged tissue containing similar cell types (see below). These two circles together exhibited a concentric organization (Fig. 3m’, n’, q’, r’) and were the precursor tissues of girdle. We term these larval tissues as the girdle field, which is composed of the outer and inner girdle field (shaded regions in Fig. 3m’, n’, q’, r’). The outer girdle field generally overlapped with the spicule-formation region during metamorphosis (compare Fig. 3m with Fig. 2k), suggesting that it contained spicule-formation cells.

**Fig. 4:**
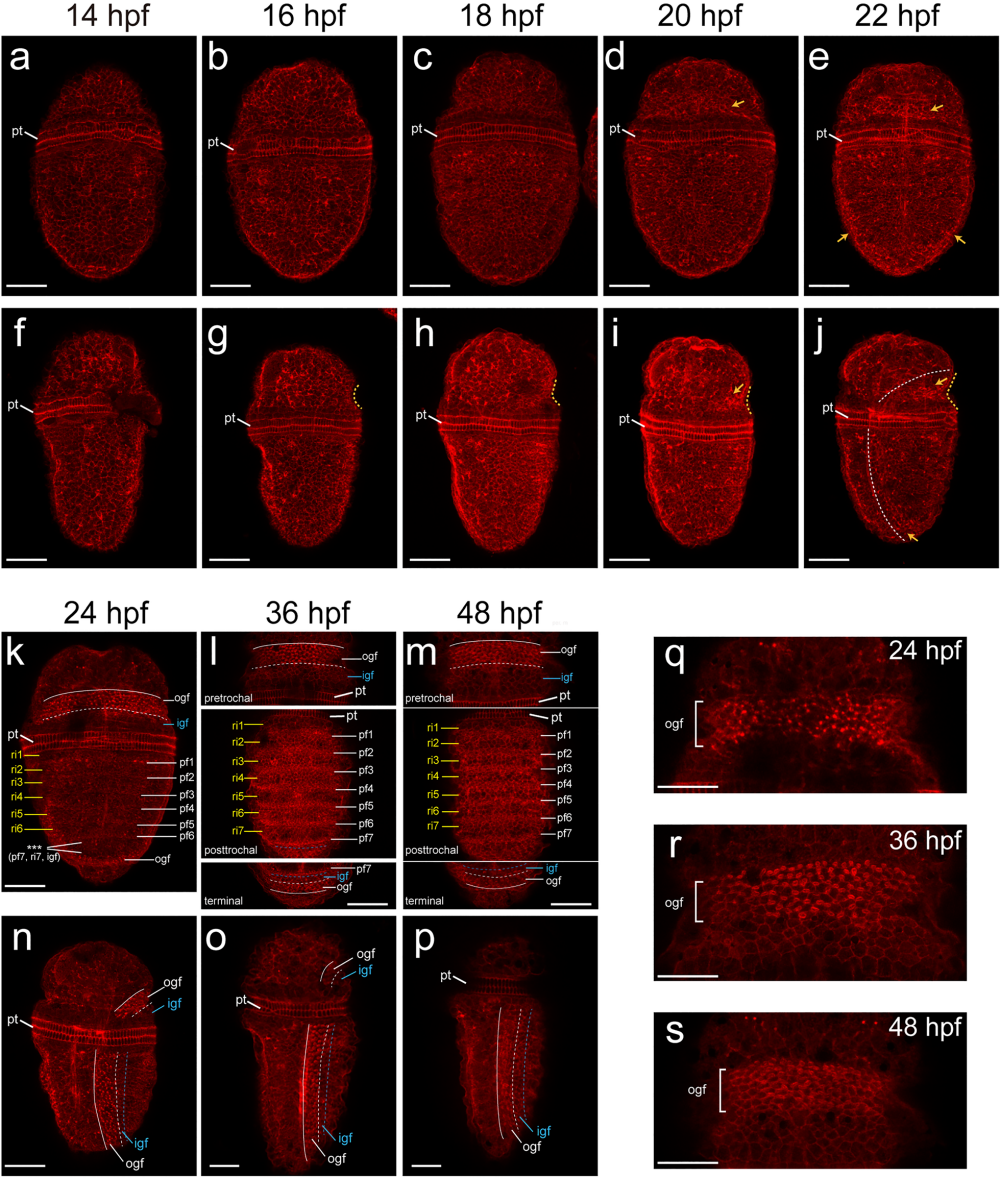
F-actin dynamics during morphogenesis of the shell field. Dorsal (**a–e**, **k–m**) and lateral (**f–j**, **n–p**) views are shown. The presumptive shell field tissues in the pretrochal region becomes invaginated at 16 hpf (dashed line in **g**). F-actin starts to aggregate in this region at 20 hpf (arrow in **d** and **i**), which then spreads to the posttrochal region (arrows in **e** and** j**). These aggregations show “spot” patterns at 24 hpf and “circled” patterns at 36 and 48 hpf (**k–m**; more details are shown in **q–s**). Plate fields and ridges are formed in the central region at 24 hpf (**k**). Stars in **k** indicates that the seventh ridge, the seventh plate field and inner girdle field are not detectable. The inner girdle field is recognizable between the outer girdle field and the central region (in the posttrochal region) or between the outer girdle field and prototroch (in the pretrochal region) (**k–p**). ri, ridge; pf, plate field; ogf, outer girdle field; igf, inner girdle field; pt, prototroch. Bars represent 50 μm

Specification in the central region of the shell field became evident at around 22 hpf, when alternative bulges and grooves were discernable (Fig. 3k). Soon at 24 hpf, most bulges and grooves were well-developed (lacking the last ones; Fig. 3l, and compare to Fig. 4k). The bulges and grooves did not emerge at the same time and their numbers could vary among samples collected at the same timepoint, indicating rapid formation. These bulges and grooves showed minor changes in later larvae (36–48 hpf; Fig. 3m, n). According to previous reports, only the grooves will produce shell plates, and they are designated plate fields [6, 11], while the bulges are called (intersegmental) ridges [11].

### F-actin dynamics during shell field morphogenesis

As SEM revealed continuous morphogenetic changes during the development of the shell field, we investigated the dynamics of filamentous actin (F-actin) to explore more details. In particular, CLSM (confocal laser scanning microscopy) 3D projections were carefully prepared to avoid influences from strong staining in larval muscles (Additional file 1: Figure S1; see Methods). F-actin was generally evenly distributed in the dorsal epithelium of early larvae (Fig. 4a–c), despite random concentrations without a clear pattern. Beginning at 16 hpf, the pretrochal tissues adjacent to the prototroch became slightly depressed, and this depression sustained till at least 22 hpf (yellow dashed curves in Fig. 4g–j). This depression indicated the development of the girdle field. Nevertheless, it was not until 20 hpf that F-actin aggregation was observed in this region (Fig. 4d, i). The F-actin aggregations became further evident at 22 hpf, and simultaneously, comparable aggregations became detectable in posttrochal cells (Fig. 4e, j). They continued to enhance at 24 hpf and outlined the specifying outer girdle field (Fig. 4k, n).

At the same time, weak but steady F-actin stripes were detected in the developing plate fields (except the last one that was not developed yet; Fig. 4k), separating by ridges devoid of such F-actin patterns. In the inner girdle field, no characteristic F-actin distribution patterns were detected, but this region could be roughly determined under CLSM as a gap between the outer girdle field and the developing ridges/plate fields (Fig. 4n). Together, all four subregions of the shell field were recognizable at 24 hpf, indicating that the larval shell field was generally established at this stage (Fig. 4k, n).

**Fig. 5:**
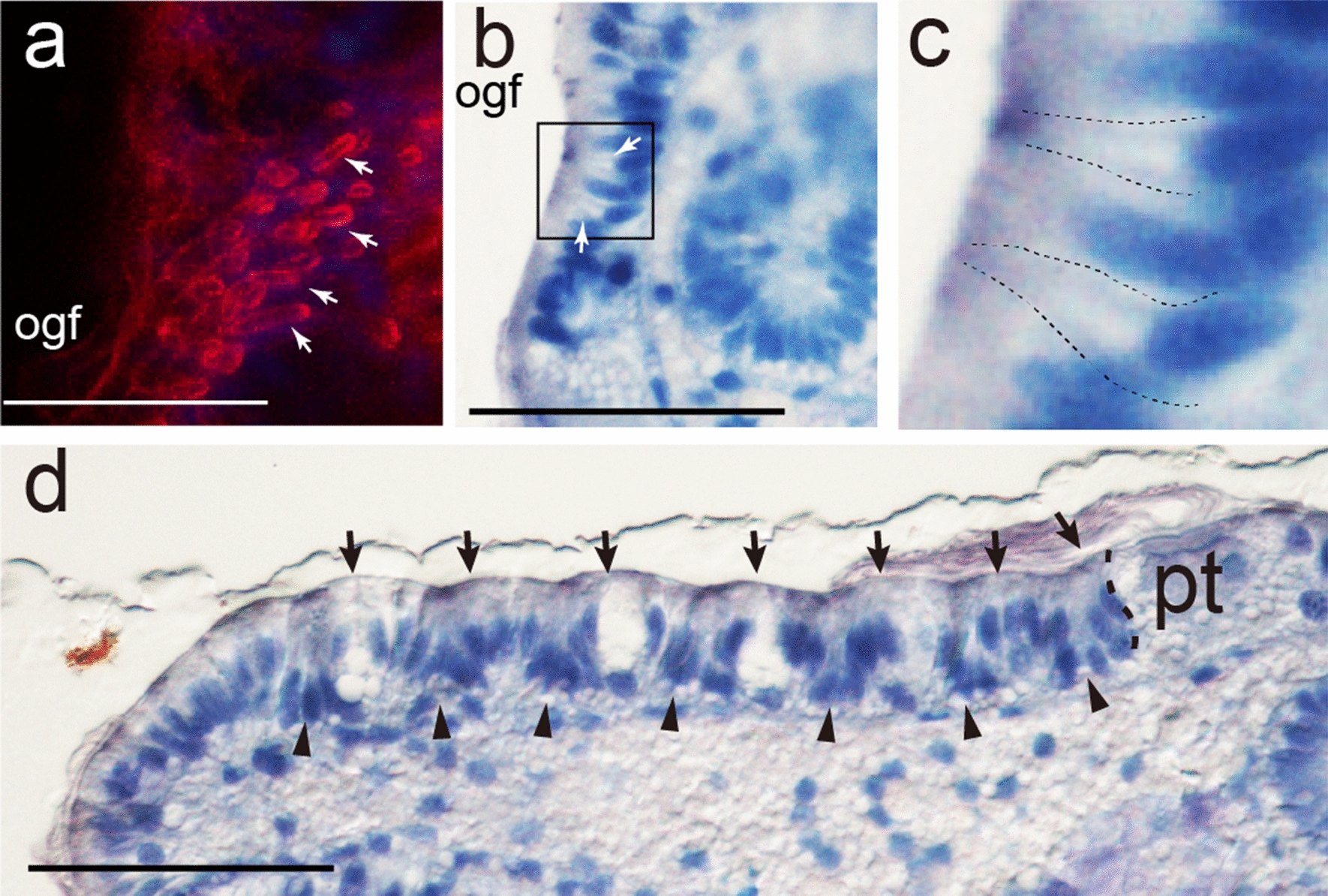
Details of the outer girdle field and the plate fields. **a**–**c** Tube-likes structure of the presumptive spicule formation cells (arrows) in the outer girdle field (ogf). **a** CLSM 3D projection showing F-actin “tubes” (arrows). **b, c** Semithin section cross the outer girdle field showing similar structures (arrows). **c** is a magnified image corresponding to the area indicated by the box in** b**, in which the two recognizable tubes are highlighted by dashed lines. **d** Longitude section showing the posttrochal region of a 48-hpf larvae, with anterior to the right and dorsal on the top. Plate fields (arrowheads) and ridges (arrows) can be recognized. Note that the cells adjacent to the prototroch (pt) are not invaginated, indicating that the first plate field does not reach this distance. Bars represent 20 μm

F-actin aggregations in the outer girdle field showed “spotted” patterns initially (from the apical view; Fig. 4q), which then transitioned into tiny “circles” (36 and 48 hpf; Fig. 4r, s). CLSM 3D projections revealed that they were actually “tubes” that inserted deeply inside the larval body (Fig. 5a). From semithin sections, similar tube-like structures could be recognized in this region (Fig. 5b, c), and we interpret these structures correspond to the F-actin tubes under CLSM. Interestingly, it was revealed that each tube was derived from a single cell with a deep nucleus (Fig. 5b, c). This type of cells were alternatively arranged with other cell types with more superficial nuclei (Fig. 5b, c), consistent with the alternative arrangement of cell populations with and without F-actin aggregations under CLSM (Additional file 1: Figure S2a–d). These structural characteristics indicate that the cells showing F-actin aggregations may be spicule-formation cells (see Discussion).

In the central region, the signals of phalloidin staining (F-actin stripes) became further stronger with ontogenesis (Fig. 4l, m), indicating continuous development of plate fields. From longitude semithin sections, the plate field cells exhibited deeply located nuclei, contrasting with the more superficial nuclei of ridge cells (Fig. 5d). CLSM optical sections revealed similar results, and further demonstrated that although having deep nuclei, the apical sides of plate field cells were actually exposed to the surface (Additional file 1: Figure S2e–i).

### *Engrailed* expression

We finally investigated the expression of the well-known shell-formation gene *engrailed* [12]. In *A. rubrolineata*, *engrailed* expression was detected in both dorsal and ventral tissues. We previously revealed that the dorsal domain of *engrailed* expression was related to shell development [15].

**Fig. 6:**
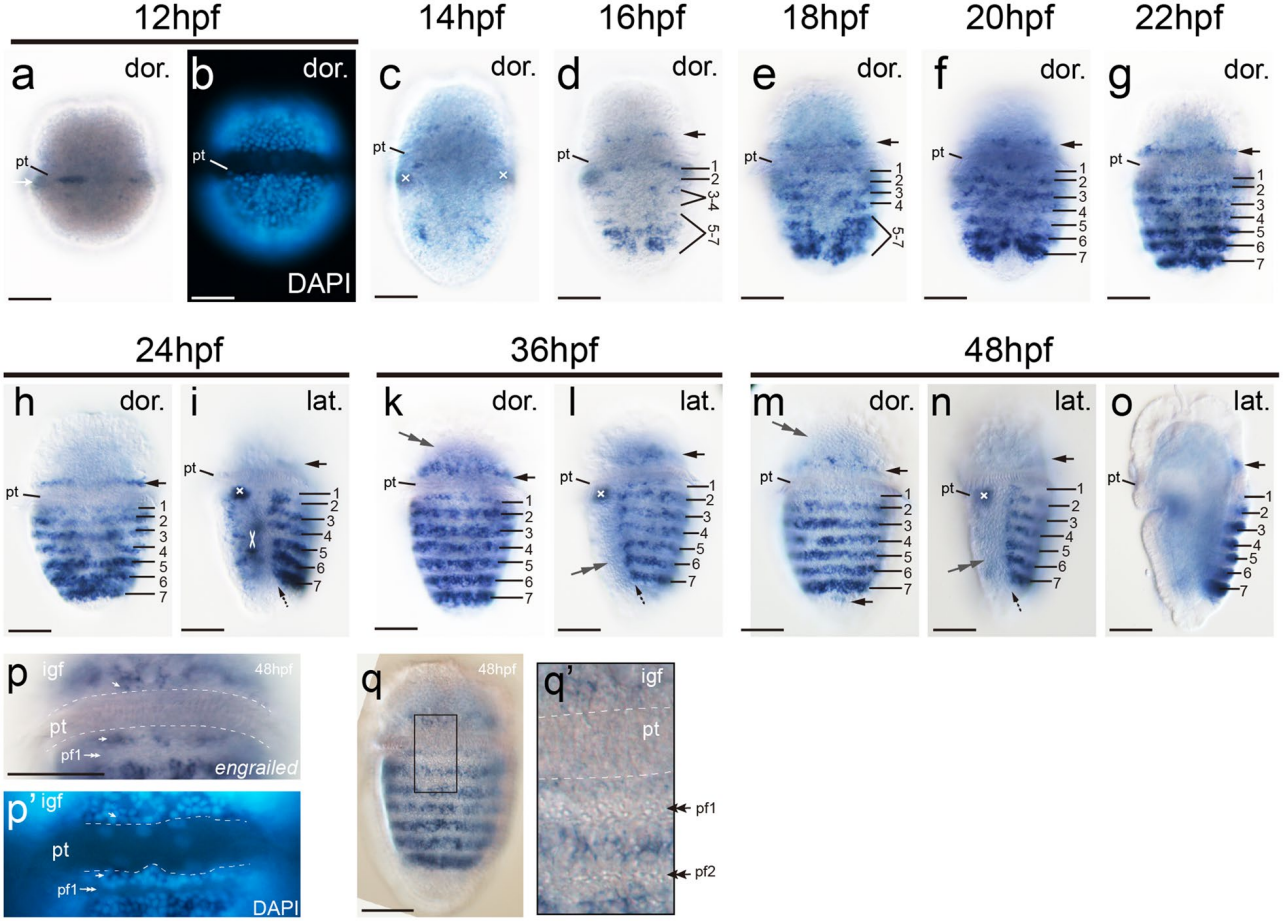
Expression of the shell formation gene *engrailed* during early development of *A. rubrolineata*. Dorsal (**a–h**, **k**, **m**) and lateral (**i**, **l**, **n**, **o**) views are shown, with the anterior on the top. In lateral views (**i, l, n, o**), dorsal is to the right. Striped expression in the posttrochal region (ridges) is indicated by numbers (1–7). Black arrows in **d–o** indicate the expression in the inner girdle field that encircles the central region showing striped expression. Expression of *engrailed* with no evident correlation with shell field development is indicated by white crosses. Particular morphological characteristics could be recognized in the outer girdle field (gray double arrows in **k–n**), which should correspond to the tiny protrusions under SEM (compare to Fig. 3q’). Panels **p** and **p’** show the *engrailed* expression adjacent to the prototroch, in which several positive cells right adjacent to the prototroch are highlighted by white arrows. Panel **q’** corresponds to the region enclosed by the black box in **q**, which shows granular structures in plate fields but not in cells adjacent to the prototroch. pf, plate field; igf, inner girdle field; pt, prototroch. Bars represent 50 μm

Dorsal expression of *engrailed* could be detected at 12 and 14 hpf, which was scattered distributed in both pretrochal and posttrochal regions and it was difficult to conclude a clear pattern (Fig. 6a). A notable fact is that evident *engrailed* expression was detected in several cells very adjacent to the prototroch (Fig. 6a, b). Major changes were observed at 16 hpf, when *engrailed* expression was evidently enhanced and covered a relatively large area of dorsal epithelium (Fig. 6d). More importantly, beginning at this stage, the dorsal *engrailed* expression showed a trend of striped expression (Fig. 6d). At 18 and 20 hpf, the striped expression of *engrailed* became much more clear and seven stripes were ultimately established in the posttrochal region later (Fig. 6e, f). It could be determined that the expression was in intersegmental ridges, and this expression pattern was sustained in subsequent development (Fig. 6k, m).

Interestingly, these seven stripes of *engrailed* expression showed different modes of ontogenesis from each other. The two most anterior ones, stripes 1 and 2, were formed individually. They were recognizable since 16 hpf and gradually established from 16 to 20 hpf (Fig. 6d–f). In contrast, the other five stripes were derived from two relatively broad expression regions. The first region was relatively faint at 16 hpf (Fig. 6d), and became stronger and split into two stripes (stripes 3 and 4) at 18 hpf (Fig. 6e). The second, more posterior region was somewhat intensely stained at 16 hpf (Fig. 6d), and gradually transitioned into three stripes in subsequent development (stripes 5–7; Fig. 6e, f). Moreover, the two *engrailed* expression regions also showed a trend of bilateral pattern along the dorsal midline, which was even detectable after the full development of five stripes (Fig. 6d–g). This is somewhat consistent with the fact that we frequently observed a middle depression throughout the posterior ridges of some larvae under SEM (Additional file 1: Figure S3).

In addition to the striped expression in the central region, a pretrochal stripe of *engrailed* expression was gradually established in early larvae (arrows in Fig. 6d–h). In the posttrochal region, the left and right marginal cells adjacent to the ridges also began to show *engrailed* expression at 24 hpf, and became evident later (dashed arrows in Fig. 6i, l, n). Ultimately, *engrailed* expression became discernable in cells posterior to the seventh ridge, and it connected the left and right marginal expression (the lower arrow in Fig. 6m). All aforementioned *engrailed* expression formed a circle, corresponding to the inner girdle field.

A close look revealed that the cells right adjacent to the prototroch, both pretrochally and posttrochally, all expressed *engrailed* (Fig. 6p, q), indicating they were not involved in shell plate formation. This is consistent with the observation under DIC microscopy that although small granular structures could be observed in plate fields, they were never observed in more anterior cells adjacent to the prototroch (Fig. 6q’).

## Discussion

Polyplacophoran shell development is important to understand the evolution of mollusks as well as their shells [2, 3, 6, 7, 18, 19]. Previous studies pay primary attention to the formation of shell plates, which actually represents relatively late stages of shell development [9, 11, 12], leaving shell field morphogenesis largely unknown. Moreover, while previous research revealed the various cell types inside the shell field and their behaviors during shell formation [2, 6, 11], efforts are required to explore the molecular aspects. Some genes are reported to be expressed in the shell field [13–17], but their correlations with particular cell types remain largely unknown.

**Fig. 7:**
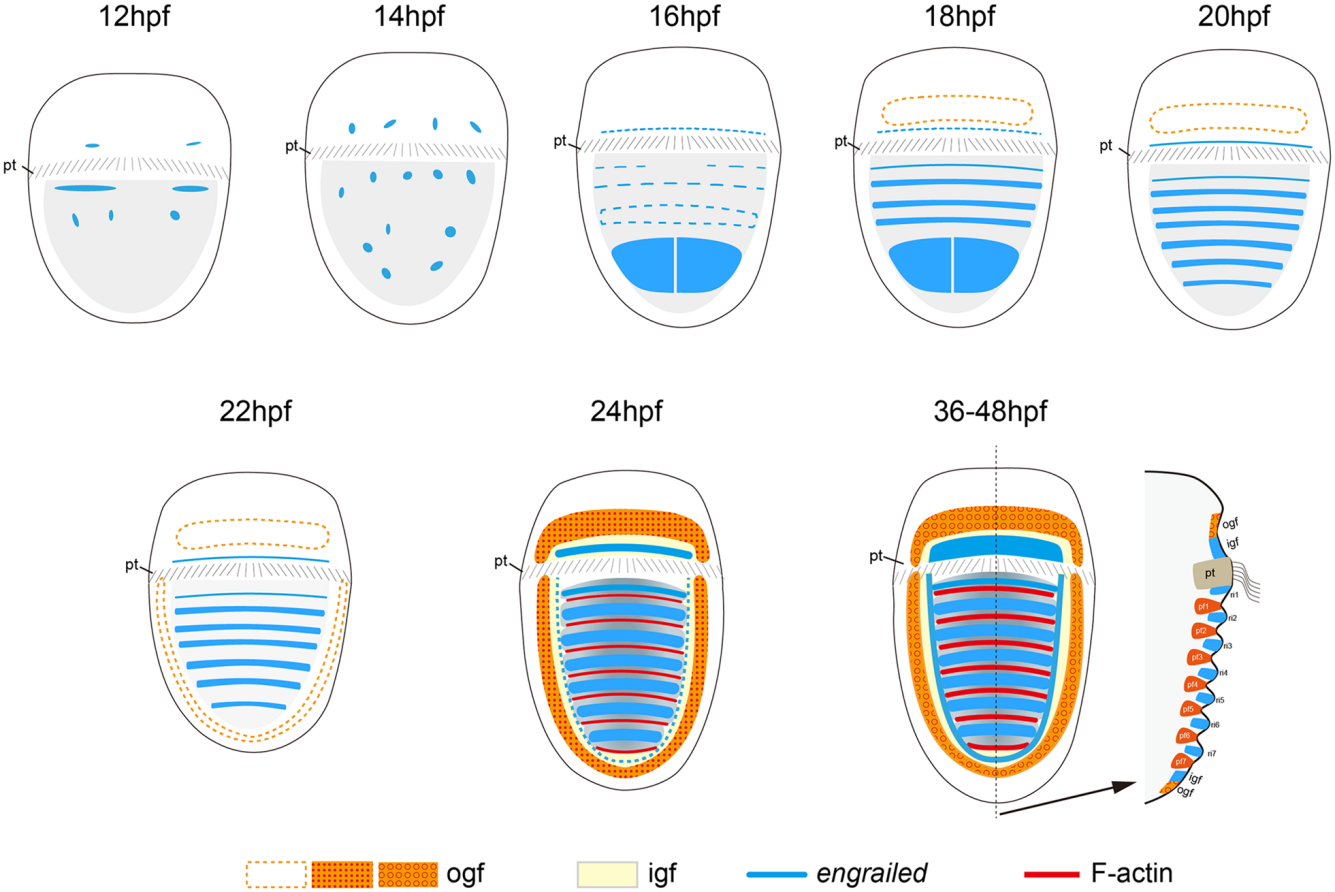
Schematic diagrams showing shell field morphogenesis in *A. rubrolineata*. Different parts of the shell field can be recognized based on gene expression patterns or aggregated F-actin. The organization of the shell field is generally established at 24 hpf, including the girdle field comprising the inner and outer girdle fields and the central region showing alternatively arranged plate fields and ridges

In the present study, we investigated morphological and molecular changes during shell field morphogenesis in the chiton *A. rubrolineata*. Previous studies reported a recognizable shell field [11] or no shell field development [10] in newly hatching larvae. We found that in *A. rubrolineata*, neither a morphologically discernable shell field, nor molecular evidence indicating its existence, could be detected in newly hatched larvae, suggesting that shell field morphogenesis occurred totally in larval stages. This feature makes *A. rubrolineata* particularly useful to study shell field morphogenesis. Combining the results of multiple approaches, we revealed relatively complicated developmental events during the process. The different patterns of F-actin aggregations in outer girdle field and plate fields, coupled with *engrailed* expression in inner girdle field and ridges, comprise the molecular patterns of different parts of the larval shell field. Schematic diagrams summarizing our findings are shown in Fig. 7.

### Development of the central region: plate fields and ridges

The serial arrangement of chiton shell plates attracts attention from researchers [6, 9–11]. In *A. rubrolineata*, we observed characteristic seven plate fields that were alternatively arranged with seven intersegmental ridges, similar to all other polyplacophorans investigated. The ontogenesis of these two types of tissues, namely, shell plate fields and ridges, are tightly correlated, indicating that they are modulated by the same regulatory signals.

One notable fact is that the plate fields exhibited significant invagination in *A. rubrolineata*, which can be reflected by much deeper nuclei compared to those of ridge cells (Fig. 5d); a similar feature is also reported in *Cyanoplax caverna* (= *Lepidochitona cinera*) [6]. In contrast, the cells of plate fields and ridges are distributed in comparable depths in *Ischnochiton rissoi* (= *Ischnochiton rissoa*) and *Cyanoplax caverna* (= *Lepidochitona caverna*) [11, 12], indicating a different situation with much reduced invagination of plate fields. Accordingly, very different morphological characteristics of *engrailed* positive cells are revealed in *A. rubrolineata* (Fig. 6o) and *C. caverna* (= *L. caverna*) [12] (Fig. 3F of the study). We cannot determine whether these different degrees of invagination are caused by variations among species or developmental stages. Indeed, evident shape changes were reported in the cells of plate fields during shell plate genesis [11]. However, given that invagination has been a common process in molluscan shell development and should affect shell formation [2, 20, 21], the apparent different degrees of plate field invagination in polyplacophorans are worthy of further investigations.

Kniprath identified four cell types (1–4) in the central region of the shell field in *I. rissoi* (= *I. rissoa*), including types 3 and 4 in the plate field producing the shell plates and types 1 and 2 in the intersegmental ridges that generate connective tissues between shell plates [11]. Type 1 cells are gland cells with goblet shapes, which are surrounded by type 2 cells with high contents of granular endoplasmic reticulum. Adjacent to them are type 3 and 4 cells with homologous but less dense cytoplasm, which consist almost exclusively of free ribosomes. Microvilli are observed in type 2–4 cells, but are never developed in type 1 cells. Similar results are revealed in *C. caverna* (= *L. caverna*), and type 1 and 2 cells are further demonstrated to express *engrailed* [12]. Our results are consistent with these reports and further revealed that F-actin aggregations marked plate fields, i.e., type 3 and 4 cells. However, we could not determine whether they marked a single or both types of these cells.

The plate fields and ridges became morphologically detectable in a very short time in *A. rubrolineata* (between 22 and 24 hpf), making it difficult to explore the details of their morphogenesis. We thus traced the dynamics of related molecules to reveal more details. The *engrailed* expression was continuously changing before the formation of morphologically discriminable ridges (16–24 hpf), suggesting relatively complicated development. The key characteristic of *engrailed* expression is that it initially showed no clear patterns (12–14 hpf), and gradually transited into striped pattern later (16–24 hpf). This mode of development seems to be different from the formation of striped gene expression of segmentation genes in vertebrates or *Drosophila*, indicating that the serial ridges (and plate fields) may be formed through different manners from those used in the segmentation of vertebrates or *Drosophila* (segmentation clock or simultaneous formation of segments [22]).

We concluded several additional characteristics regarding *engrailed* expression in ridges (and thus the development of ridge and plate field). First, different stripes of *engrailed* expression experienced varied modes of changes. In particular, stripes 1 and 2 formed individually, but other five stripes were derived from two expression areas, each of which transitioned into two (no. 3–4) or three (no. 5–7) stripes later. These different modes of genesis should provide useful clues to explore the underlying regulatory mechanisms. For instance, the formation of anterior and posterior *engrailed* stripes (ridges) may be regulated by varied mechanisms, reminiscent of the varied regulatory mechanisms underlying the formation of anterior and posterior somites in amphioxus [23]. Second, the formation of these seven stripes of *engrailed* generally follow an anterior-to-posterior pattern (Fig. 6d–g). This pattern, as well as the fact that the expression levels of *engrailed* were apparently higher in posterior ridges (Fig. 6g, h), may indicate the involvement of a morphogen with graded activities along the anterior–posterior axis. Finally, the two posterior *engrailed* expression regions showed a bilateral pattern in relatively early stages. This may indicate the existence of a central signaling center along the dorsal midline.

Unlike the dynamic *engrailed* expression, we observed very limited changes in the F-actin aggregations of the developing shell field. The aggregation of F-actin in this region was only detected after the formation of morphologically detectable plate fields (24 hpf). It then enhanced with the invagination of plate fields (compare Fig. 4k, l), indicating a potential role of actomyosin networks in this invagination event, while a structural role of actin frameworks cannot be denied. Moreover, although we did not detect molecular markers for the plate fields in earlier stages (before 24 hpf), they may be revealed in future investigations.

### The chiton shell field includes a marginal region

The girdle (or perinotum) of adult chiton encircles the shell plates and develops spicules on it. It is proposed that the epithelium of the girdle is not so different from that producing the shell plates [6]. From this perspective, it is reasonable to take girdle development as a part of shell formation. On the other hand, although the girdle precursor was noticed previously, it was frequently paid less attention than the plate fields [11, 12]. Our results clearly revealed that the girdle precursor of *A. rubrolineata* experienced complicated development comparable to that of ridge/plate fields and that it contained different cell populations arranged in a regular pattern. Since spicules are essential to explore molluscan shell origin [6–8], we propose to emphasize the girdle precursor and suggest a specific term for it. Here, we picked “girdle field” for simplicity and clarity, and to distinguish between larval and adult tissues. This term should be used to describe the larval tissue of polyplacophorans that (1) will develop to girdle after metamorphosis, and (2) contains cells of certain degrees of specification related to girdle development. The girdle field of *A. rubrolineata* comprised two cell populations forming concentric circles, namely, the inner and outer girdle field (Figs. 3 and 7). As also shown in *Lepidochitona caprearum* (= *Middendorffia caprearum*) [11], the girdle field was separated into pretrochal and posttrochal parts by the prototroch.

The outer girdle field likely contained spicule-formation tissues that exhibited superficial tiny protrusions and F-actin aggregations (“spots” or “tubes” at different stages) but lacked *engrailed* expression. This is consistent with the previous observation that only the larval girdle without *engrailed* expression develop spicules [12]. It was reported that the polyplacophoran spicule was formed within a deep invagination of a papillar cell, which had a collar formed by its apical part [6]. We found that the F-actin “tubes” in the outer girdle field were deeply inserted into the larval body and were likely derived from a single cell (Fig. 5a–c), indicating they should correspond to the apical collars of spicule-formation cells mentioned previously. Moreover, although the spicule-formation cells already showed superficial tiny protrusions when the F-actin “tubes” emerged, there was no morphological characteristics for these cells in the earlier stage (24 hpf), and they can be only recognized based on the “spotted” F-actin patterns. This fact can facilitate identifying spicule-formation genes during the early specification of spicule-formation cells.

The demonstration of the correlation between characteristic F-actin aggregations and spicule formation would contribute to several evolutionary issues concerning aculiferan spicules. First, as two representatives of extant aculiferan mollusks, the common feature of polyplacophorans and aplacophorans is the ability to develop spicules, and thus spicule development should contain important information of aculiferan evolution. Although almost the same process of spicule formation is suggested in two aculiferan clades [6], molecular evidence is required to certify this notion. Current research concerning aplacophoran spicule development reveal relatively few molecular data [24–27], and it would be intriguing to explore whether aplacophoran spicule formation also exhibited similar F-actin dynamics as we revealed. Second, some researchers speculate that molluscan spicules have a common evolutionary origin with the chaetae/setae of annelids and brachiopods, thereby being greatly informative to infer spiralian evolution [8]. The potential homology of these sclerotized structures has been explored based on the fine structure of the sac that generate them as well as related genes/molecules [28]. However, from current reports, we could not discern specific F-actin aggregations in annelid or brachiopod chaetal sacs [29–31]. On the other hand, as we have shown in Additional file 1: Figure S1, such a correlation may be relatively subtle and could be masked by the strong staining in muscular tissues. Further CLSM analysis with a high resolution and devoid of influences from muscles may contribute to exploring this issue. Investigating the expression and function of chaeta-formation genes in *A. rubrolineata* is also required.

Consistent with the report in *C. caverna* (= *L. caverna*) [12], we found circular expression of *engrailed* corresponding to the gap between the outer girdle field (marked by F-actin aggregations) and the central region. We interpreted these circularly organized cells to be a part of the girdle field, and termed them as the inner girdle field. Although the roles of these cells are not fully elucidated, it is reported that they are not involved in spicule development and contain some secretory cells [11, 12]. Moreover, when taking the *engrailed-*expressing tissue in the shell field as a whole, it is attempting to propose that the *engrailed* expression seems to define the boundary of shell plates: that in the inner girdle field demarcates the entire region bearing all shell plates, and the expression in ridges delineates each plate. A similar idea regarding the role of *engrailed* in determining developmental boundaries was proposed previously [12].

The common expression of *engrailed* in intersegmental ridges and inner girdle field indicates similarities between related cells. This is consistent with the report that type 1 and 2 cells are distributed in both ridges and girdle of *I. rissoi* (= *I. rissoa*) [11]. Similarly, common F-actin aggregations were revealed in presumptive spicule-formation cells in the outer girdle field and those of the plate fields (despite the different patterns), indicating some common features between these sclerotization-related cells. It is also notable that in both the girdle field and the central region, the tissues showing *engrailed* expression and F-actin aggregations are adjacent to each other but never intermixed.

### Pretrochal contribution to shell plate formation?

If accepting the development of girdle to be a part of shell development, the involvement of pretrochal tissues in shell development could be confirmed by the fact that the girdle field contains both pretrochal and posttrochal tissues [11, 12]. However, this speculation does not contribute to the debate concerning whether the pretrochal region contribute to the formation of the first shell plate [9, 11]. Since we did not investigate shell formation in metamorphosis, our results provide no direct evidence to this question. However, if shell plates are indeed secreted by the cells of plate fields, our results seem to indicate no pretrochal contribution to shell plate formation in *A. rubrolineata*. We found that the tissues right adjacent to the prototroch, in both pretrochal and posttrochal regions, all expressed *engrailed* (Fig. 6p, q) and thus should not be involved in shell plate formation. The first plate field was distributed posteriorly to these tissues (Fig. 5d), indicating the first shell plate should be posttrochal.

### Molluscan shell evolution: shell fields, shell plates and spicules

The shell field represents a key node of shell development, and it should provide useful clues to explore essential questions concerning molluscan shell evolution, i.e., whether conchiferan and polyplacophoran shell plates are homologous [3, 6, 19]. Conchiferan shell development has been investigated in various lineages, especially with the aid of molecular markers, such as gene expression and endogenous enzyme activities [20, 32–38]. It is clear that conchiferan shell fields show a concentric (“rosette”) pattern, with different cell populations distributed in varied distances from the center. More importantly, the shell plate is only formed in the central region of the shell field [2, 20, 38–41]. These features are also revealed in the polyplacophoran *A. rubrolineata*. Despite interrupted by the prototroch, the girdle field enclosed the central region, where the (seven) shell plates would develop. The girdle field itself also showed a comparable concentric organization, comprising two circles of F-actin “spots/tubes” (outer girdle field) and *engrailed* expression (inner girdle field).

The common expression of *engrailed* in the girdle field is another shared feature between polyplacophorans and conchiferans [12, 15, 35, 37, 42]. Similarly, comparable expression of *pax2/5/8* is observed in the girdle field of the chiton *Acanthochitona crinita* [16] and the margin of larval mantle in a gastropod [43]. Therefore, despite the very different shell plates they produce, a key feature between polyplacophoran and conchiferan shell fields is the existence of a marginal region of shell field and the common expression of particular genes in the tissue, while the shell plate(s) will develop in the central region they surround.

However, it is still too early to say that the aforementioned data support the homology of polyplacophoran and conchiferan shell fields. Researchers revealed different cell lineages for polyplacophoran and conchiferan shell fields, arguing against such homology [3, 19, 44–46]. In fact, the common feature of aculiferan mollusks is the development of spicules but not shell plates. If assuming that a shell plate could be evolved when multiple spicule-formation cells join together (amalgamation) [6, 7], it is more favorable to speculate that the common organization of polyplacophoran and conchiferan shell field may be the prerequisite of placing the sclerotization cells in the central region, which underpins the common evolution of shell plates in these two lineages. The highly similar shell fields themselves, however, may be the result of convergent evolution. Alternatively, there is evidence indicating that the last common ancestor of aculiferans may have both shell plates and spicules [3, 7]. In this respect, the likelihood may somewhat increase regarding the potential homology between conchiferan and polyplacophoran shell fields, given that they share the many characteristics mentioned above.

Given the well-supported phylogeny of mollusks comprising of Aculifera and Conchifera [47, 48], polyplacophorans are more evolutionarily close to aplacophorans than conchiferans. Thus, the similarities between the shell field of polyplacophorans and conchiferans bring an intriguing question, that is, whether spicule-formation tissues in aplacophorans share some common characteristics with polyplacophoran (and conchiferan) shell fields? Due to the lack of shell plates, a shell field does not exist in aplacophorans. Nevertheless, here we call the larval epithelium related to spicule development as the aplacophoran shell field for simplicity, given the accepted homology between aplacophoran and polyplacophoran spicules. Seven rows of sclerotization cells/spicules in the dorsal larval epithelium of two aplacophorans [24, 27] resemble the seven serially arranged plate fields in polyplacophorans, suggesting shared features of aculiferan shell fields. However, it is not known whether aplacophoran shell fields also possess a marginal region with a concentric organization. Another line of evidence is that *gbx* is common expressed in the epithelium responsible for spicule/shell development in a aplacophoran, a polyplacophoran and a bivalve conchiferan [17].

Taken together, it could be concluded that some common features of shell fields are revealed among aculiferans, between polyplacophorans and conchiferans, or between aculiferans and conchiferans, but they are far from sufficient to certify any issues regarding the homology of shell fields, that of shell plates, or that of shell plates and spicules. Compared to the extensive researches in conchiferans, studies on the development of aculiferan sclerotized structures are limited, and further investigations are required to make better comparisons to explore molluscan (shell) origin and evolution.

Finally, it is notable that the development of the eighth shell plate and aesthete represents other essential aspects of polyplacophoran shell development. Given that the polyplacophoran shell field contains only the anlagen for the first seven shell plates, other manners of development may be employed for the eighth one. The sensory aesthete canal system refers to a branching canal system embedded in polyplacophoran shell plates. It is believed to be evolutionarily important due to the observation of similar structures in the shell plates and/or sclerites of ancient aculiferans and their presumptive stem lineages [7, 49, 50]. However, since the development of the eighth shell plates and aesthete occur after metamorphosis, they were not investigated in the present study. These would be interesting issues for future studies.

## Conclusions

Four types of tissues were recognized from the shell field of the polyplacophoran mollusk *A. rubrolineata*, namely, the inner and outer girdle fields showing a concentric organization and the centrally located, alternatively arranged plate fields and ridges. Molecular patterns are revealed for each type of tissues, including common F-actin aggregations in the outer girdle field and the plate fields (with different patterns), and *engrailed* expression in the inner girdle field and the ridges. These results provide a detailed description regarding the structure of the polyplacophoran shell field on both molecular and cellular levels. The dynamics of different molecules revealed the ontogenesis of related tissues and provide clues for exploring the underlying regulatory mechanisms. Further studies are required to elucidate the roles of each type of cells, as well as the mechanisms modulating their specification and organization, which will help understand the formation and evolution of polyplacophoran shells and spicules.

## Methods

### Animals and larval culture

The adults of *A. rubrolineata* (Lischke, 1873) were collected from intertidal rocks in Qingdao, China. After transferred to the lab, each individual was placed in a 100-ml plastic cup filled with fresh seawater. In reproductive seasons (June–August), a proportion of individuals spawned within approximately 3 h after the transferring, and each type of gametes was thus collected. Sperm was added to oocyte suspension for artificial fertilization, and zygotes were cultured in filtered seawater (FSW) at 25 °C in an incubator. The developmental stages were referred to as hpf.

Trochophore larvae hatched after 8.5 hpf. A few abnormal larvae were neglected in most circumstances; when their numbers could not be neglected, healthy larvae were collected from the upper half of the water column at around 10 hpf. The larvae older than 60 hpf could be induced to metamorphose by supplying plastic sheets coated with the algae collected from the rock that their parents inhabited. We found that the timepoints to start metamorphosis were very different for different individuals. This prevented us from investigating the development during and after metamorphosis in detail, but indeed allowed us to collect a few such samples. Live samples at varied developmental stages were recorded using an Olympus CKX53 inverted microscope.

At desired developmental stages, the larvae were anesthetized by adding 1 M MgCl_2_, fixed in 4% paraformaldehyde (PFA) (1 × PBS, 100 mM EDTA, 0.1% Tween-20, pH 7.4) or 2.5% glutaraldehyde, and stored in methanol or PBSTw (1 × PBS, 0.1% Tween-20, pH 7.4), as described previously [38].

### Scanning electron microscopy

Samples were gradually dehydrated to ethanol, and successively transferred to a mixture of ethanol and isopentyl acetate (*v*/*v* = 1:1, once) and isopentyl acetate (twice). They were then submitted to critical-point drying (with liquid CO_2_), coated by gold, and observed using a scanning electron microscope (Hitachi S-3400N).

### Semithin sectioning

Samples fixed by glutaraldehyde and stored in PBSTw were stained with hematoxylin and eosin before subsequent manipulations. After washed with PBSTw, stained samples were gradually dehydrated to ethanol and successively transferred to acetone and resin (EPON 812). After curing, samples embedded in resin was sectioned into 1 μm sections. Although we stained the samples with two dyes, we found eosin staining was washed out in subsequent treatments, the samples were thus only stained dark blue.

### Phalloidin staining

Fixed samples stored in PBSTw were successively treated with PBSTx (1X PBS plus 0.5% TritonX-100) and 0.1% BSA in PBSTx for 5 min each. Then, the samples were stained in 0.1 μM TRITC (tetramethylrhodamine)-conjugated phalloidin (Solarbio, cat. no. CA1610) at 4 °C overnight. After washing with PBSTw, the samples were mounted in glycerol and observed under a confocal laser scanning microscope (ZEISS LSM 710). In the negative control group, phalloidin was not included in the staining solution, and this yield no signals, indicating no autofluorescence of the larvae that may influence with the results.

In CLSM, Z-stack projections were applied in most circumstances. When doing this, the optical sections were carefully selected to not include the deep sections containing very strong staining in the larval muscle. This strategy helped reveal very important structures, such as the F-actin aggregations in plate fields (Additional file 1: Figure S1).

### Whole mount in situ hybridization

Whole mount in situ hybridization using a probe targeting the shell-formation gene *engrailed* was performed as described previously [15]. In brief, a pair of specific primers (forward: AAGTTCTCTGGCATCATTCGTAG, reverse: GTCTATCTCCTCATCGTCCCTTC) were used to amplify the cDNA fragment of *engrailed* of *A. rubrolineata*. During PCR, the T7 promoter sequence (taatacgactcactataggg) was included in the reverse primer. After electrophoresis and cleaning, the PCR product was used as the template to generate the digoxin-labeled probe through in vitro transcription.

Samples stored in methanol were rehydrated to PBSTw. For acetylation, rehydrated samples were transferred to TEA buffer (1% triethanolamine in PBSTw), and then treated with 0.3% acetic anhydride in TEA buffer for 5 min. After that, acetic anhydride was added to a final concentration of 0.6% and the samples were incubated for additional 5 min. After wash with PBSTw, samples were digested with 50 μg/ml protease K in PBSTw for 20 min at room temperature. Post-fixation was performed by incubating samples with 4% PFA for 2 h at room temperature. After pre-hybridization in hybridization buffer (50% formamide, 5 × SSC, 50 μg/ml heparin, 500 μg/ml yeast tRNA, 0.1% Tween-20, pH 6.0) at 65 °C for 2–5 h, specimens were transferred to hybridization buffer containing 1 ng/μl denatured probe, and incubated at 65 °C overnight. Post-hybridization washing was also performed at 65 °C, including successively washing with washing solution (50% formamide, 2 × SSC, 0.1% Tween-20; 30 min × 2), 2 × SSCT (2 × SSC and 0.1% Tween-20; 15 min) and 0.2 × SSCT (0.2 × SSC and 0.1% Tween-20; 30 min × 2). Then, the specimens were rinsed in PBSTw at room temperature and incubated with the blocking solution (PBSTw containing 0.5% blocking reagent (Roche)) at room temperature for 2 h. Antibody staining was performed by incubating the samples with blocking solution containing 1/5000 alkaline phosphate-conjugated Fab fragments of a sheep anti-digoxigenin antibody (Roche) at 4 °C overnight. After extensively washing with PBSTw, the specimens were incubated with NBT/BCIP (Roche) for color development. After that, samples were post fixed with PBSTw containing 3% PFA and 1% glutaraldehyde to avoid the fading of staining in subsequent manipulations. Stained samples were mounted in glycerol and observed under a Nikon 80i microscope.

A sense probe was used as the negative control. Due to the limit of samples at some developmental stages, and since *A. rubrolineata* larvae were frequently used in the in situ hybridization experiments in our lab and signals were never observed in control groups, we only investigated control groups using samples at 20, 36 and 48 hpf in the present study, and no staining was observed.

### Reproducibility

All experiments were repeated at least twice and they generated the same results. In each assay, we examined multiple individuals from the same batch of samples: at least 10 in SEM and phalloidin staining (under CLSM) and at least 20 in in situ hybridization. All revealed highly consistent results. Very few samples (no more than 5%) showed somewhat inconsistent results due to obviously abnormal or slowed development (likely caused by bad oocyte quality or delayed fertilization, respectively).

## Supplementary Information

**Additional file 1: Figure S1.** 3D projections using different subset of optical sections. The two figures are derived from the different subsets of the same CLSM file. As shown in the top right inserts, panel a is derived from the subset of optical sections that does not include muscle staining, while all sections were used in panel b. It is clearly that the inclusion of muscular tissues in the 3D projection (b) strongly affects the recognition of F-actin stripes in the shell field (compare to a). **Figure S2.** Organization of different parts of the shell field revealed by CLSM. Serial optical sections revealed the details of outer girdle field (a–d) and central region (ridge and plate field; e–j). In a–d, superficial nuclei were only observed for the cells lacking F-actin aggregations, which are alternatively arranged with the cells possessing the F-actin tubes. In e–j, despite the deep locations of the nuclei in plate fields (arrows) compared to those of ridges (arrowheads), the apical sides of plate field cells were expose to the surface. **Figure S3.** Dorsal view of a 24-hpf larva. In this larva, evident depression could be observed accross the central region of posterior ridges along the midline, as highlighted by the arrow.

## Data Availability

All data generated during this study are included in this published article.

## Abbreviations

hpf: Hours post fertilization
F-actin: Filamentous actin
SEM: Scanning electron microscopy
CLSM: Confocal laser scanning microscopy
FSW: Filtered seawater
PFA: Paraformaldehyde
