# Supplementary material for: Shell field morphogenesis in the polyplacophoran mollusk Acanthochitona rubrolineata
Source: EvoDevo. 2023 Apr 6;14:5. doi: 10.1186/s13227-023-00209-9 (PMC10080879; doi:10.1186/s13227-023-00209-9)
Supplement: Supplementary file 1 — Additional file 1: Figure S1. 3D projections using different subset of optical sections. The two figures are derived from the different subsets of the same CLSM file. As shown in the top right inserts, panel a is derived from the subset of optical sections that does not include muscle staining, while all sections were used in panel b. It is clearly that the inclusion of muscular tissues in the 3D projection (b) strongly affects the recognition of F-actin stripes in the shell field (compare to a). Figure S2. Organization of different parts of the shell field revealed by CLSM. Serial optical sections revealed the details of outer girdle field (a–d) and central region (ridge and plate field; e–j). In a–d, superficial nuclei were only observed for the cells lacking F-actin aggregations, which are alternatively arranged with the cells possessing the F-actin tubes. In e–j, despite the deep locations of the nuclei in plate fields (arrows) compared to those of ridges (arrowheads), the apical sides of plate field cells were expose to the surface. Figure S3. Dorsal view of a 24-hpf larva. In this larva, evident depression could be observed accross the central region of posterior ridges along the midline, as highlighted by the arrow. [file 13227_2023_209_MOESM1_ESM.pdf]

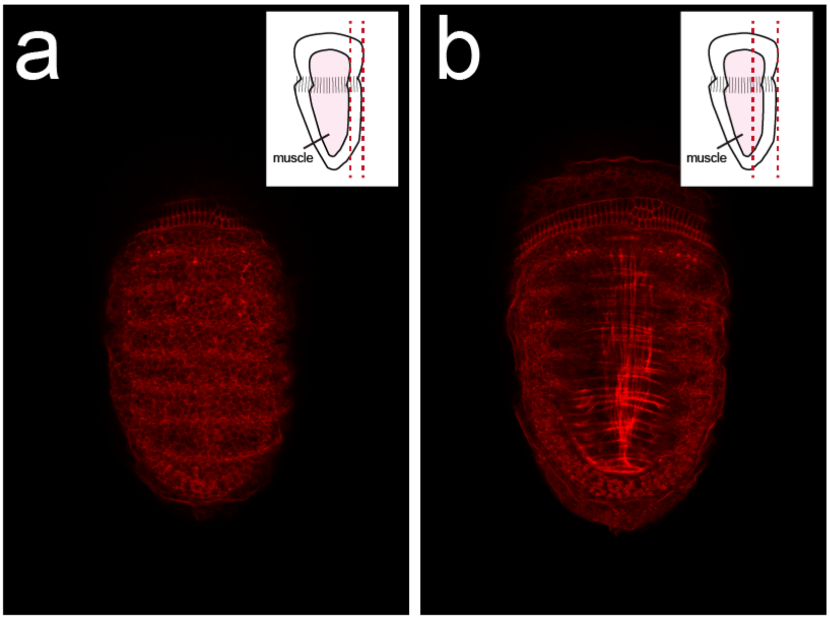

Figure S1. 3D projections using different subset of optical sections. The two figures are derived from the different subsets of the same CLSM file. As shown in the top right inserts, panel **a** is derived from the subset of optical sections that does not include muscle staining, while all sections were used in panel **b**. It is clearly that the inclusion of muscular tissues in the 3D projection (**b**) strongly affects the recognition of F-actin stripes in the shell field (compare to **a**).

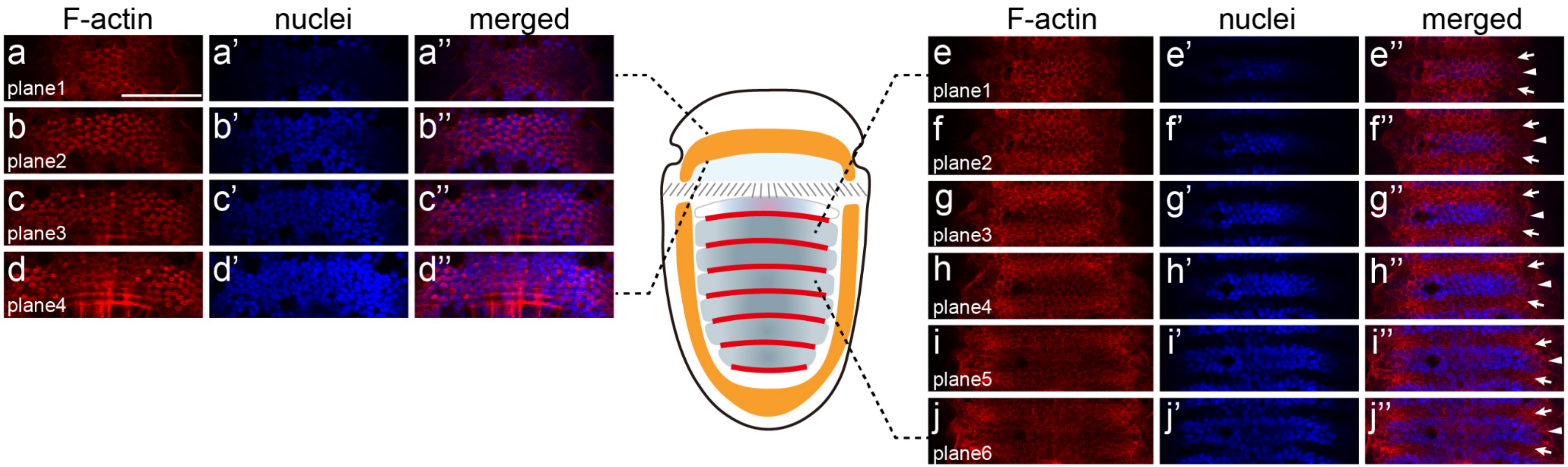

Figure S2. Organization of different parts of the shell field revealed by CLSM. Serial optical sections revealed the details of outer girdle field (**a-d**) and central region (ridge and plate field; **e-j**). In **a-d**, superficial nuclei were only observed for the cells lacking F-actin aggregations, which are alternatively arranged with the cells possessing the F-actin tubes. In **e-j**, despite the deep locations of the nuclei in plate fields (arrows) compared to those of ridges (arrowheads), the apical sides of plate field cells were expose to the surface.

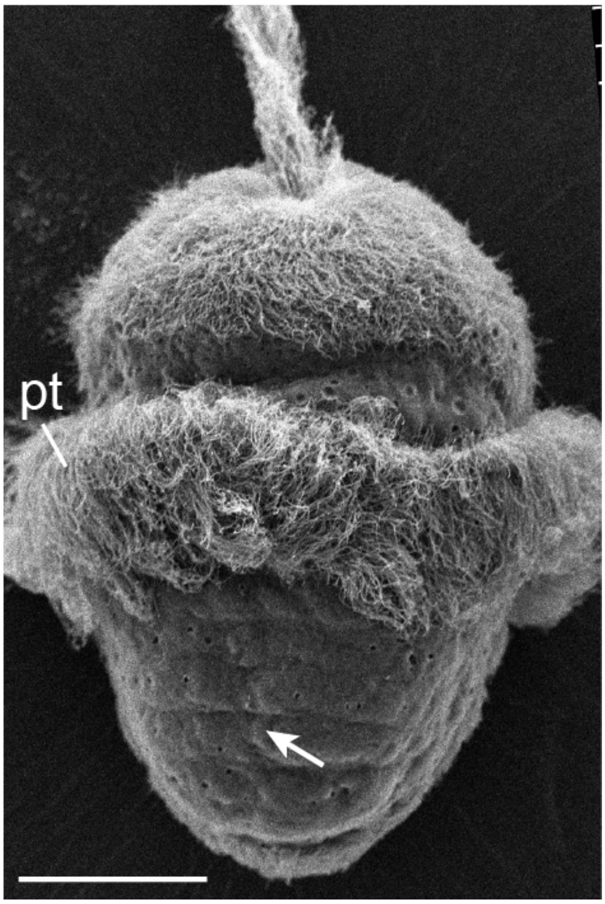

Figure S3. Dorsal view of a 24-hpf larva. In this larva, evident depression could be observed accross the central region of posterior ridges along the midline, as highlighted by the arrow.
